# Covalently-Linked Hyaluronan versus Acid Etched Titanium Dental Implants: A Crossover RCT in Humans

**DOI:** 10.3390/ijms20030763

**Published:** 2019-02-11

**Authors:** Saturnino Marco Lupi, Arianna Rodriguez y Baena, Clara Cassinelli, Giorgio Iviglia, Marco Tallarico, Marco Morra, Ruggero Rodriguez y Baena

**Affiliations:** 1 Department of Clinico-Surgical, Diagnostic and Pediatric Sciences, Dental Clinic, University of Pavia, P.le Golgi 2, 27100 Pavia, Italy; arianna_rodriguez@hotmail.it (A.R.yB.); ruggero.rodriguez@unipv.it (R.R.yB.); 2 Nobil Bio Ricerche srl, V. Valcastellana 26, 14037 Portacomaro, Italy; ccassinelli@nobilbio.it (C.C.); giviglia@nobilbio.it (G.I.); mmorra@nobilbio.it (M.M.); 3 Private Practice, V. Vincenzo Ussani 86, 00151 Roma, Italy; me@studiomarcotallarico.it

**Keywords:** dental implants, surface modification, hyaluronan, clinical trial

## Abstract

Biochemical modification of titanium surfaces (BMTiS) entails immobilization of biomolecules to implant surfaces in order to induce specific host responses. This crossover randomized clinical trial assesses clinical success and marginal bone resorption of dental implants bearing a surface molecular layer of covalently-linked hyaluronan in comparison with control implants up to 36 months after loading. Patients requiring bilateral implant rehabilitation received hyaluronan covered implants in one side of the mouth and traditional implants in the other side. Two months after the first surgery, a second surgery was undergone to uncover the screw and to place a healing abutment. After two weeks, the operator proceeded with prosthetic procedures. Implants were evaluated by periapical radiographs and the crestal bone level was recorded at mesial and distal sites—at baseline and up to 36 months. One hundred and six implants were positioned, 52 HY-coated, and 48 controls were followed up. No differences were observed in terms of insertion and stability, wound healing, implant success, and crestal bone resorption at any time considered. All interventions had an optimal healing, and no adverse events were recorded. This trial shows, for the first time, a successful use in humans of biochemical-modified implants in routine clinical practice and in healthy patients and tissues with satisfactory outcomes.

## 1. Introduction

Ever since the pioneering studies of Professor Brånemark, osseointegration of titanium implant fixtures has been recognized as an interfacial event [1]. The clinical practice of implant dentistry has been based on the intimate apposition of newly formed bone tissue to the titanium surface. A great deal of literature has been devoted to the relationship between titanium surface properties and new bone formation [2,3,4]. The primary physical-chemical variables, titanium oxide surface chemistry, and implant surface topography dictate relevant surface parameters—such as surface charge and wettability—and have been deeply investigated in relation to cellular events leading to peri-implant bone regeneration [5,6,7,8,9,10,11]. The understanding of such relationships prompted the clinical evolution of titanium implants from the original turned to present day micro- and nano-rough surfaces [12,13,14]. 

Parallel to the growth and widespread acceptance of dental implantology, rising knowledge framed relevant interfacial biological events within a broader picture [15,16,17,18]. Cellular mechanisms leading to new bone formation and soft tissue healing, as well as inflammatory response leading to loss of supporting soft and hard tissue, are mediated by biological molecules and relevant signaling. The presentation of biomolecular cues, rather than the comparatively rough inorganic chemistry of titanium, seems a reasonable highway towards the evolution of better and innovative implant surfaces [19]. Accordingly, surface engineering of medical devices has long since been involved with the immobilization of a wide range of biomolecules to medical materials surfaces [20,21]. Biochemical modification of titanium surfaces (BMTiS) was defined by Puleo and Nancy [22] as the immobilization of proteins, enzymes, or peptides with the purpose of inducing specific cell and tissue responses using critical organic components of bone to affect tissue response. BMTiS are generally based on surface modification either by peptides, ECM proteins, or polysaccharides, all from animal and vegetal sources [23,24,25,26,27,28,29,30,31,32,33,34]. Despite a huge number of studies and promising in vitro and pre-clinical results [35], no practical application exists so far, and clinical performances of present-day dental implants are still dictated by the titanium/host tissue interface and mostly based on stimulation of cell behavior by surface topography.

This work presents the results of the first clinical trial involving bio-molecular modification of titanium implant surfaces. Implant fixtures used in the present trial bear a surface nano-layer (a few nm thick) of covalently-linked hyaluronic acid or hyaluronan [while its common acronym is HA, it is indicated as HY in the present paper to avoid any risk of confusion with the widely used inorganic HA (hydroxyapatite)-coatings]. This means that they present a complex macromolecular chemistry and not the relatively simple inorganic chemistry of titanium surfaces at the implant/tissue interface.

**Figure 1:**
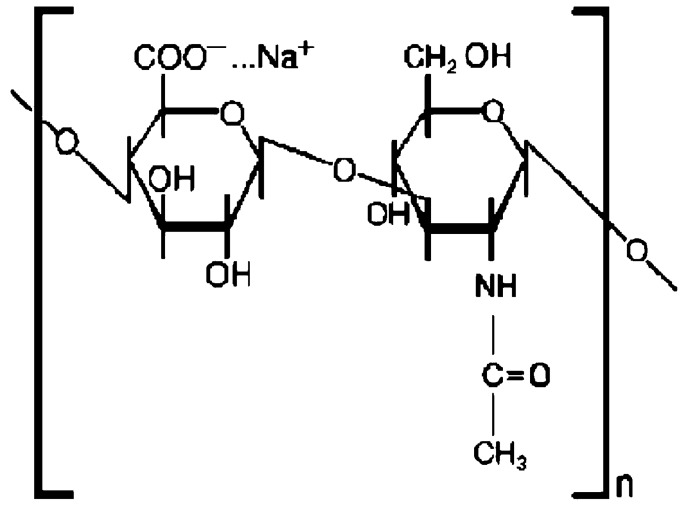
The repeating unit of Hyaluronan (HY).

HY (Figure 1) is a linear polysaccharide consisting of the repeating disaccharide unit *N*-acetyl-d-glucosamine-d-glucuronate linked by β1-4 and β1-3 linkages. It is involved with a huge number of cellular processes [36,37,38,39]. 

Contrary to more complex glycosaminoglycans, HY is not sulfated, and it does not occur as part of a proteoglycan linked to a protein carrier. Significant interest in HY as a biomaterial and in biomaterials surface modification exists [40]. HY in medicine is mostly exploited because of its physical properties (hydration, viscosity, space filling), or by taking advantage of the hydration-promoted ability to reduce non-specific adhesion. Growing knowledge on HY as a key molecule in the regulation of many cellular processes involved with wound healing and tissue regeneration suggests that even more opportunities lie in the exploitation of its specific biological and bioactive properties [41,42,43].

Several literature reports indicate the potential interest of HY in BMTiS. Concerning bone regeneration, since the fifties it has been known that considerable HY is synthesized in the early stages of callus formation during the repair of fractured long bones [44]. Iwata and Urist [45] found that large amounts of HY were secreted when implants of decalcified bone underwent remineralization as bone. Bernard and coworkers presented studies aimed at developing “a foundation for the use of HY as a superior carrier for osteotropic substrates, even as HY acts to enhance osteogenesis due to its own molecular structure” [46]. Their in vitro studies using fetal calvarial cells and bone marrow osteogenic stem cells show that osteogenesis in vitro is significantly enhanced by HY 30–160 kDa, while high Mw HY (550–1300 kDa) shows weak inhibitory effects compared to the control. Zou and coworkers reported that 800 kDa HY added to bone marrow stromal cells cultured in vitro accelerates cell proliferation, increases alkaline phosphatase activity and osteocalcin gene expression, and that HY interacts with BMP-2 to generate direct and specific cellular effects [47]. Ito and coworkers showed that locally applied 900 kDa HY has a positive effect in bone ingrowth in Titanium fiber mesh implant in rats [48]. According to Cho, HY shows a positive effect in early bone consolidation in distraction osteogenesis [49]. HY based scaffolds aid in the regeneration of cartilage and bone defects in tissue engineering applications. The hypothesis of an active role played by local HY delivery upon scaffold degradation was suggested [50]. Zhao et al. [51] investigated the role of molecular weight and concentration of HY on the proliferation and osteogenic differentiation of rabbit bone marrow-derived stem cells in vitro. Factorial analysis indicated that molecular weight (MW) and concentration had an interactive effect on alkaline phosphatase mRNA expression (*p* < 0.05). HY of higher MW and higher concentration promoted bone formation. Regarding the in vivo studies on HY-coated implants, Aebli et al. [52] did not find bone growth increase in tests involving a sheep model. It should be noted that in the quoted study, the water-soluble HY was simply applied from solution to hydroxyapatite-coated implants without any intervening chemical bond to prevent rapid wash off [40]. An in vivo study on surface-engineered titanium implants bearing instead of a covalently-linked HY molecular surface layer in a four week rabbit model showed improvement of both bone to implant contact and bone ingrowth by hystomorphometry, while mechanical testing and evaluation of interfacial bone micro-hardness confirmed a faster bone maturation around HY coated implants [53]. Based on these and other encouraging pre-clinical results, the present study was conducted to investigate the clinical potential of HY covalently-linked implants and to set a starting point for future developments. The main goal was to confirm, in clinical practice and adopting objective clinical evaluation criteria, that “it is possible to do without the titanium surface chemistry”. Once this point is set in routine clinics, pathways to actual exploitation of biomolecular signaling properties in compromised or challenging cases can be explored. 

The paper presents first a detailed investigation of HY-coated titanium implants surfaces and relevant uncoated controls by SEM, energy dispersive X-ray spectroscopy (EDX), and X-ray photoelectron spectroscopy (XPS). Results of the clinical trial are then presented and discussed.

This split-mouth randomized clinical trial is aimed at assessing the clinical success and marginal bone resorption of dental implants bearing a surface molecular layer of covalently-linked hyaluronan in comparison with traditional sand-blasted and etched titanium implants up to 36 months after loading.

## 2. Results

### 2.1. Scanning Electron Microscopy/EDX Analysis

**Figure 2:**
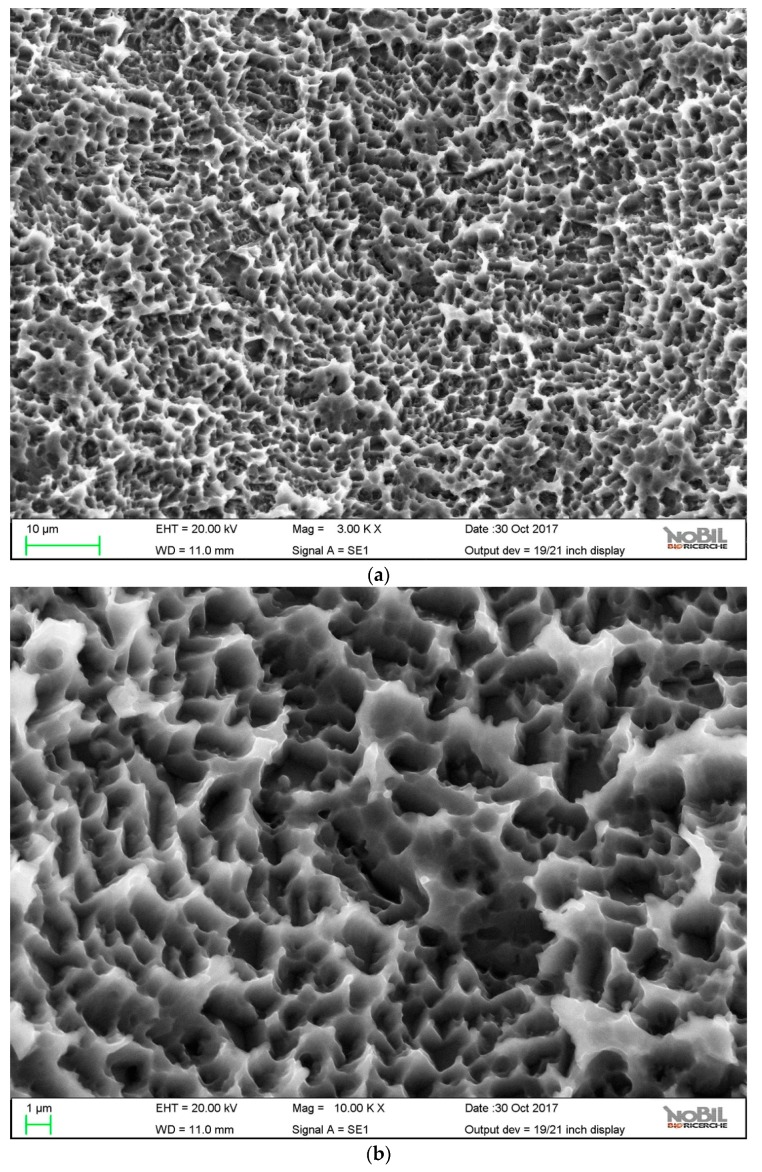
SEM images of the control implant surface, (**a**) 3000×; (**b**) 10,000×.

**Figure 3:**
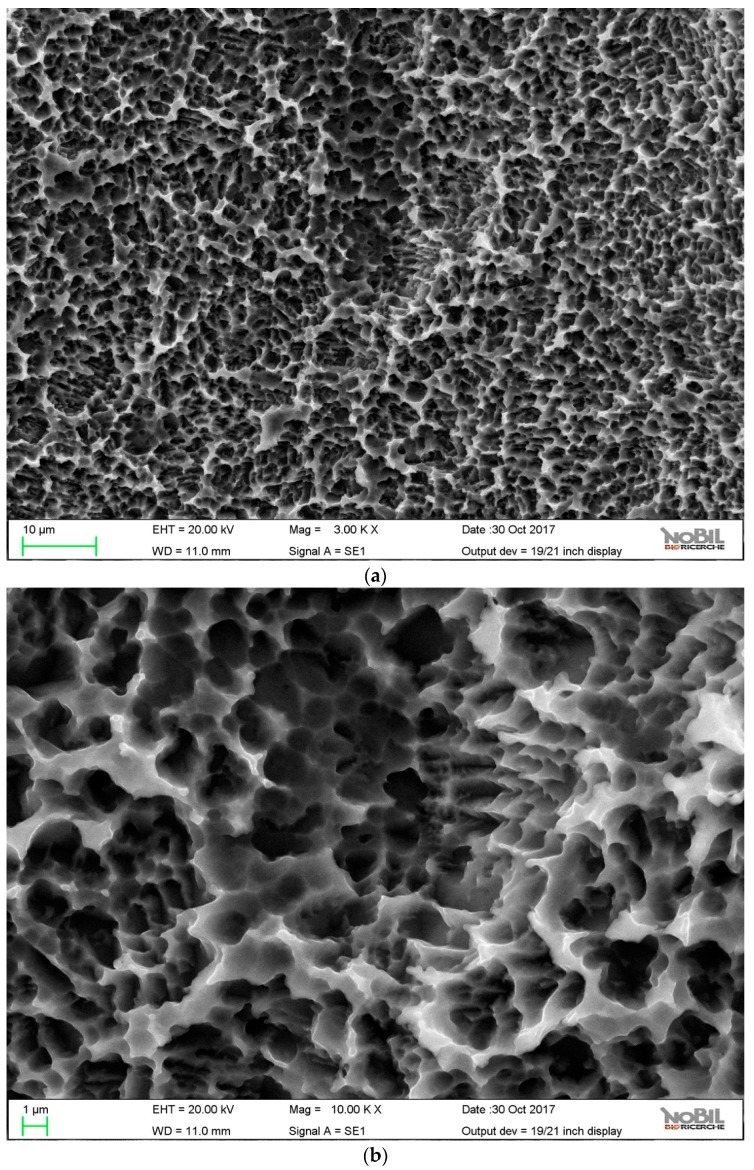
SEM images of the hyaluronan- (HY) coated implant surface, (**a**) 3000×; (**b**) 10,000×.

The surface topography of control and HY-coated implants was evaluated by SEM. Figure 2 and Figure 3 show obtained results at 3000× and 10,000×.

Both samples showed the typical microtopography of doubly acid etched surfaces—a microrough surface where the distance between peaks was of the order of the micrometer. As reported in the literature, a peak distance lower than the typical cell length can stimulate cell behavior, thus promoting accelerated osteogenesis [54,55]. No evidence of the HY coating was observed, even at 10,000×. Surface linking of HY involved just molecular layers, whose thicknesses were approximately a few nanometers at most [40]. At this level of magnification, it was not detected over the microrough landscape provided by the doubly acid etched titanium surface. Table 1 reports the roughness parameters according to ISO 4287 that were obtained by the stereo-SEM reconstruction of the surface topography obtained from 2000× images, as described in the Materials section. A 900 µm path length and an 80 µm cut-off wavelength were used. No significant difference between samples was observed.

**Figure 4:**
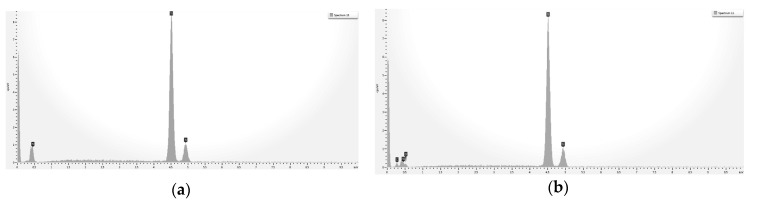
EDX spectra of control (**a**) and HY-coated (**b**) titanium implant.

Figure 4 shows the energy dispersive X-ray spectroscopy (EDX) spectra obtained from the two samples. Both of them showed a very strong signal from titanium. In the case of the HY-coated implant, small but significant peaks due to carbon and oxygen were detected as well, suggesting the presence of an organic surface layer. 

### 2.2. X-Ray Photoelectron Spectroscopy

**Figure 5:**
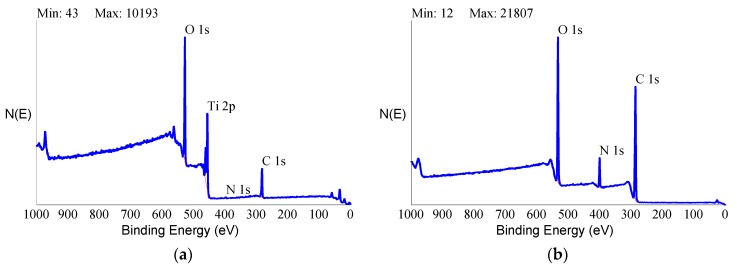
X-ray photoelectron spectroscopy (XPS) wide-scan spectra of control (**a**) and HY-coated (**b**) titanium implant.

XPS wide-scan spectra of control and HY-coated dental implants used in the present clinical trial are shown in Figure 5. 

The control implant showed the typical composition of titanium surfaces, yielding signals due to photoemitted electrons from core levels of the expected elements: titanium, oxygen (because of the native titanium oxide surface layer), and carbon (due to surface adsorption of ubiquitous carbon-containing compounds from the atmosphere and nitrogen). The overall surface composition, reported in Table 2, was in good quantitative agreement with data from the literature. It showed no unexpected elements or contamination, and its C/Ti ratio was indicative of excellent surface cleanliness [7,56]. 

**Figure 6:**
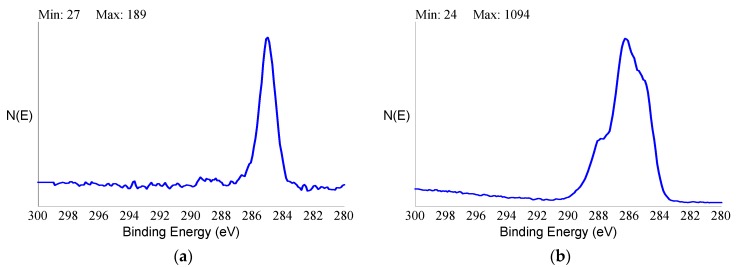
XPS high-resolution C1s peak of control (**a**) and HY-coated (**b**) titanium implant.

The XPS spectrum of the HY-coated implant, on the other hand, showed a completely different picture. First and foremost, there was no signal from titanium at all. The only elements detected in the nanometer-thick sampling depth probed by XPS were the basic elements of organic chemistry—oxygen, carbon, and nitrogen. The elemental ratio reported in Table 2 was consistent with typical polysaccharides stoichiometry, showing a high O/C ratio. Perusal of the literature confirmed that the detected surface stoichiometry was consistent with the reported composition of covalently-linked HY through aminated spacers [57]. Further clues were offered by the sensitivity to the carbon chemical environment provided by the high-resolution peak of C1s photoelectrons. Figure 6 shows C1s peaks of the tested control and HY-coated dental implants. 

**Figure 7:**
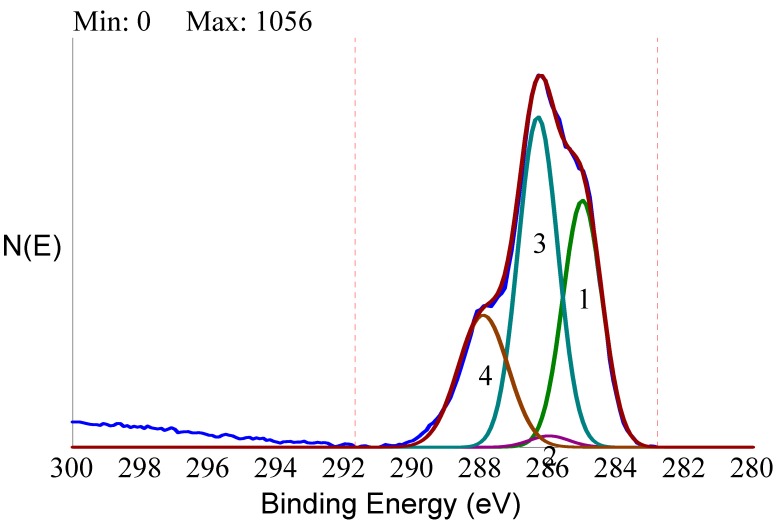
Curve fitting of high-resolution C1s peak of the HY-coated titanium implant by the four components expected from the molecular structure of hyaluronan.

In the former case, the peak was relatively simple and symmetrical, confirming that it was mostly due to C-C and C-H functionalities from adventitious hydrocarbons. The C1s peak of the HY-coated implant was much broader and clearly contained different components. De-convolution of the C1s peak of the HY-coated implant following general XPS practice [58] is shown in Figure 7. 

The experimental curve was fitted according to literature indication [59] by four components located at 285.00 eV (C-C, C-H), 286.15 eV (C-N), 286.50 eV (C-O), and 288.10 eV (C=O, N-C=O, and O-C=O functionalities). Peak de-convolution showed that the carbon chemical environment at the HY-coated titanium implant surface was dominated by the carbon single bond oxygen functionality typical of polysaccharides, and that the experimental C1s peak contained all features expected from the molecular structure of HY. Taken together, these data convincingly showed that the outermost nanometers of the HY-coated implant surface presented to the external environment the molecular cues stemming from the HY repeating unit, which was a completely different chemical nature when compared to the titanium oxide surface of the conventional control implant. 

### 2.3. Clinical Trial

From 8 April 2013 to 17 October 2014, 106 implants were positioned in 30 patients (demographic data in Table 3). 

During surgery, no differences were observed in regards to the insertion and stability between the two types of implants (test and control), nor were differences observed in the post-operative visits in regards to the indices of inflammation and wound healing. The healing was uneventful in all of the patients in the variability of the surgical situation, and no adverse events whatsoever were recorded. Only 102 implants in 29 subjects out of the 106 implanted were loaded and taken as baselines for X-ray values (mesial and distal). Two implants (one control and one HY coated) in two different patients failed and were removed. In both cases, the mucosa appeared edematous and bleeding, indicating signs of an infection. In one case, the implant was not replaced, and since no matching controls were available, the subject (Subject N°34) was excluded from the efficacy analysis. In the second subject, the failed implant was relocated in the same place after two months, reaching healing and tissue stabilization. Therefore, the subject was maintained in the statistics. One subject with two implants (one control and one HA) dropped out between the 12 months and the 18 months follow up visits due to a desire for pregnancy and therefore the unethical use of X-ray procedures. Results are summarized in Table 4. 

The non-inferiority analysis did not show any significant differences between the HY- and the control- in terms of mesial and distal bone resorption at any time point.

## 3. Discussion

In the present clinical trial, a widely adopted microrough titanium surface and the same surface further modified by a covalently-linked nanolayer of hyaluronan were compared in terms of clinical success in routine clinical practice.

Analytical data confirmed the exquisitely superficial nature of the surface modification process that was adopted. The analytical signal captured in EDX analysis stemmed from a surface volume that was a few micrometers deep [60]. Thus, within the EDX sampling depth, the signal by the nanometers-thick HY surface layer was diluted within the micrometer-thick analytical layer, and the resulting spectrum contained convoluted contributions from the underlying Ti implant and the overlying HY molecules in roughly a 1000:1 ratio. As a consequence, the elements composing the surface layer were barely detectable. For this reason, a more surface-sensitive analytical technique, namely XPS, was conducted to fully appreciate the biomolecular modification of the HY-coated implants. Contrary to EDX analysis, the physics of photoelectrons escaped from solids endowed XPS with just a few nanometers of sampling depth, consequently providing chemical information on the outermost atomic layers of materials [58]. For this reason, XPS was extensively used in the surface analysis of dental implants [7,61,62,63].

Despite the widely different surface chemistry, objective optimal healing was observed for both groups, and no differences were detected in the clinical outcome for all tested parameters. Far from being uneventful, the described results suggested some important reflections. From a chemical-physical point of view, it would be difficult to imagine two more different surface structures—the titanium surface (in particular, the outermost few nanometers of oxidized titanium) presented a hard, impervious interface to the host tissue, whose chemical behavior was controlled by titanium oxide interfacial chemistry, as widely described in many scientific papers [7,8,61]. On the contrary, the HY-coated implant aqueous interface was diffuse, soft, and hydrated [64]. Its chemistry stemmed from the molecular details of the *N*-acetyl-d-glucosamine-d-glucuronate repeating unit. Shortly, one of them (the control arm) belonged to inorganic chemistry. The HY-coated surface belonged instead to organic biomacromolecular chemistry. Despite being two worlds apart, no significant difference was detected in the tested clinical variables, and both of them led to successful osseointegration and clinical success. The first significant reflection from the data of the present work is that the titanium surface chemistry is not necessary to achieve a clinically satisfactory load-bearing capacity by a titanium dental implant.

The previous point could be interpreted as a no-effect of surface chemistry on osseointegration. As long as the implant surface is inert and no disturbance is introduced in the bone healing mechanism—that is, as long as no toxic or irritating compounds are released in the evolving new bone matrix independent from specific details of surface chemistry—clinically effective osseointegration will occur. If this is the case, no advancement is expected by BMTiS over conventional surfaces, and the sole opportunity to direct tissue response is through surface topography.

The last sentence falls short when compared to existing scientific evidence gathered from animal data, which shows direct effects of interfacial chemistry on bone healing in terms of peri-implant bone volume, bone to implant contact, or gene expression by peri-implant bone cells [35,53,65,66]. In the present clinical trial, a patient’s selection and surgery addressed comparatively routine clinical practice. The control arm, involving a state-of-the-art doubly acid etched microrough surface, obviously provided optimal healing and clinical success. Endpoint variables aimed at general clinical evidence expectedly confirmed that both the well-known titanium interfacial chemistry and the biologically relevant HY molecular cued direct cellular events involved with peri-implant tissue healing towards proper clinical response.

HY is a key molecule in many tissue regeneration processes; it is involved with most of the mammalian cells’ healing mechanisms [67,68,69] in a concentration and size-dependent way. The permanent linking of HY to materials surfaces avoids quick wash-off of the water soluble HY and aims at providing these regenerative properties at the peri-implant interface, as confirmed by several in vivo evidences. It is not clear yet how surface-immobilized HY compares with HY in solution in terms of the effect of the hindered conformational freedom on the multiple ligand-receptor interactions required to trigger a biological response [40]. It would be of interest to check the stimulation of bone tissue regeneration at machined interfaces of hybrid implants, the effect on soft tissue healing in the transmucosal section of tissue level implants, or the control of inflammatory responses of periodontal patients [70] and relevant clinical implications. The positive evidence supplied by the present clinical trial in standard practice opens the path to comprehensive investigation of the merits of BMTiS in clinical implantology by further finely-targeted clinical trials.

A last observation involves the relationship of present results with in vitro investigation of dental implant surfaces. A widely adopted approach involves the “adhesion and growth” paradigm, meaning that prospective surface structures are screened in terms of effects on adhesion and growth of osteoblast cells—the faster and more extensive the surface colonization by cells, the better the properties. Against this view, HY-coated surfaces are notoriously anti-adhesive in vitro [64,71], meaning that they prevent cell adhesion of a number of cell lines, including osteoblasts. Several applications of HY-coated surfaces are based on tissue-anti-adhesive properties. In vitro tests of present clinically successful HY-coated implants would provide unsatisfactory results if judged according to the “adhesion and growth” paradigm. Yet, clinical evidence as supplied by the present trial indicates that they are fit for the intended use. Far from being inconsistent, this evidence simply indicates the complexity of the peri-implant environment as opposed to the comparatively simple biological environment of in vitro tests. In clinics, osteoblasts do not adhere and grow onto the implant surface. Rather, they come to a complex peri-implant milieu after the blood clot and relevant blood cells, inflammatory cells, and the sequela of cytokine and growth factors are released in the initial stage of inflammation and healing [72]. Rather than the direct effect of the interfacial HY (or of any surface-linked biomolecular layer) on osteoblasts, it is the effect of molecular signaling on the evolution of the peri-implant biochemical environment that directs clinical outcome and that holds the potential merits of BMTiS in clinics.

In summary, the clinical trial did not record any significant difference between the HY- and the C-group in terms of clinical success and marginal bone resorption. In the present study, for the first time in clinics (to the author’s knowledge), dental implants with a biomolecular nanolayer on the surface showed a behavior similar to the commercial pure titanium one. This study presents the limits to having a follow up limited to 36 months. The duration of the follow up was chosen because in this span of time, the HY layer should be completely integrated within the newly formed interfacial tissue. Further studies should be conducted to evaluate the long-term success of HY-implants. Present data build up the ethical basis for the investigation into the merits of HY-coated implants in more challenging and compromised cases where signaling and regenerative properties encoded within the HY molecular structure could play a role that is presently not supplied by the comparatively rough titanium surface chemistry.

## 4. Materials and Methods

### 4.1. Titanium Implant

The fixture used in the clinical trial was a CE marked titanium grade 4, internal hexagon, doubly acid etched implant (Ornaghi Luigi & C, Brugherio, Italy). The implant fixture was further coated by covalently-linked HY by Nobil Bio Ricerche (Nobil Bio Ricerche srl, Portacomaro, Italy) through a proprietary process, as described in the Results section. Covalent-linking prevented the rapid wash-off of the water-soluble HY molecules, degradation by hyaluronidase, and release. The thickness of the coating, as discussed in the following sections, was a few nanometers and did not modify the nominal dimensions. The control was the same implant not coated with covalently-linked HY.

### 4.2. Surface Characterization

#### 4.2.1. Scanning Electron Microscopy

The surface topography of the implants was evaluated by SEM. Analysis was conducted using an EVO MA 10 SEM (Carl Zeiss Microscopy GmbH, Jena, Germany). The electron acceleration voltage was maintained at 20 kV with the working distance between 10 and 12.5 mm. These parameters are reported in the images, along with level of magnification (MAG) and the kind of detector utilized (Signal A = SE1 or CZ BSD).

Images were acquired in both conventional mode (Signal A = SE1) and in backscattered electron mode (Signal A = CZ BSD), allowing improved contrast between different chemical elements.

Roughness was evaluated quantitatively by stereo-SEM (SSEM) using dedicated software to convert conventional SEM images into three-dimensional data (Mex 6.0, Alicona Imaging, Raaba/Graz, Austria). A stereo-pair was built by the acquisition of the same field of view at zero and after five degrees of eccentric tilting. Following the principles of stereoscopic vision, the stereo pair was transformed into a three-dimensional reconstruction of the surface by the quoted software, providing height profiles values used as input data for the calculation of roughness parameters according to ISO 4287. The stereo-pairs were built from 2000× images taken from three randomly selected areas for each implant. 

#### 4.2.2. X-ray Photoelectron Spectroscopy (XPS)

XPS analysis was performed using a Perkin Elmer PHI 5600 ESCA system (PerkinElmer Inc., Waltham, MA, USA). The instrument was equipped with a monochromatized Al anode operating at 10 kV and 200 W. The diameter of the analyzed spot was approximately 500 micrometers, and the analyzed depth was about 5 nanometers. The base pressure was maintained at 10–8 Pa. The angle between the electron analyzer and the sample surface was 45°. Analysis was performed by acquiring a wide-range survey spectra (0–1000 eV binding energy) and detailed high-resolution peaks of relevant elements. Quantification of elements was accomplished using the software and sensitivity factors supplied by the manufacturer. High-resolution C1s peaks were acquired using pass energy of 11.75 eV and a resolution of 0.100 eV/step.

### 4.3. Clinical Trial

The study “Blind Comparison of Covalently-Linked Hyaluronan versus Control-Dental Implants in a Randomized Crossover Clinical Investigation” is a post market clinical follow-up according to MEDDEV 2.12-2 rev 2 January 2012 conducted at the Dental Service, Department of Clinical, Surgical, Diagnostic, and Pediatric Sciences, University of Pavia, Pavia, Italy. The clinical trial was conducted according to the ISO 14155-11 and the Good Clinical Practice Guidelines (GCP). The study was carried out following the rules of the Declaration of Helsinki, as revised in 2013 and approved by the Ethical Committee of the University of Pavia on 13 December 2012. On 28 February 2013, the initiation of the study was communicated to the Italian Ministry of Health with the reference number 000017. All subjects gave their informed consent for inclusion before they participated in the study. The primary objective of this non-inferiority, crossover, fixed-size, and single-center trial was to assess the dental implant success (survival rate) of HA coating implants in comparison with control implants at one year. The secondary objective was to assess marginal bone resorption of HA coating implants in respect to traditional titanium implants measured at 3, 6, 12, 18, 24, and 36 months. To test the non-inferiority hypothesis of the HY-coated implant in respect to control implants, the null hypothesis was that the HY-coated implant resulted in a lower survival rate and a higher marginal bone loss in respect to the control implant. The P level of significance was set at 0.05. Patients with bilateral partial or full edentulism requiring implant rehabilitation were enrolled; inclusion and exclusion criteria are shown in Table 5 and Table 6.

In this study, concealment and randomization were implemented centrally by an operator (G.O.) unrelated to the surgical team, to the clinical examiner, and to the statistician. After manufacturing, implants were packaged in consecutively numbered boxes. Each box contained six implants, three with the HY surface (marked with A or B) and three with the control surface (marked with B if the HY implants were marked with A, or marked with A on the contrary). All of the implants were 4 mm in diameter and were 8, 10, and 12 mm long. The two surfaces were macroscopically indistinguishable. The assignation of the label (A or B) to the surfaces was randomly assigned in each box by a computer random number generator. Data were communicated to the statistician, omitting the surface characteristic. The list of randomization was concealed until after statistical analysis was concluded. Due to the split-mouth design of the study and the randomization process, the allocation ratio was 1:1. No block restrictions were applied. 

#### 4.3.1. Surgical Procedure

During surgery, implant(s) with one surface were placed in one side of the mouth and implant(s) with the other surface were placed in the other side of the mouth. When a patient required placement of the number of implants equal in both sides of the mouth, each couple of implants came out from a different box. When the number of implants required in one side of the mouth was greater than the other side, implants were chosen among those available in the opened boxes. If the odd implants were not available in the opened boxes, no experimental implants were positioned. This procedure was implemented after protocol approval because the surgical team signaled the problem of re-operating in patients with a different number of edentulous sites in the two sides of the mouth. Even though it was not necessary because randomization was implemented before surgery, the implant label choice for the sides of the mouth was randomized by a coin toss. The surgical protocol of installing the implants was well documented and consisted of a full thickness mucosal flap elevation, the preparation of the implant site, the installation of the implant with the prosthetic platform (that is, the edge of the collar) at the level of the bone crest, the positioning of the surgical cover screw, and the closing of the flap with sutures [73,74,75,76,77,78,79,80]. The surgery was done under coverage of antibiotics (2 g of amoxicillin and clavulanic acid per os one hour before surgery, followed by administration of 1 g after 6 and 18 h). The post-operative instructions indicated rinses with chlorhexidine 0.12% × three times a day from the day after surgery and for 30 days and control of the pain with anti-inflammatory drugs as needed. The removal of the sutures was carried out from the tenth to the fourteenth day.

The second stage of surgery was conducted after two months, both in the mandibular and in the maxillary arch. After about two weeks, the operator proceeded with the prosthesis installation. During follow-up visits, normal hygienic procedures were carried out when needed [81].

#### 4.3.2. Investigation Hypothesis or Pass/Fall Criteria

The primary objective of the present study was to assess the success of HY coated implants in comparison with control implants after one year. The implant is considered successful (primary endpoints) if: (A) the implant is still present without any sign of mobility and (B) there is no evidence of radiolucency by means of periapical X-rays and (C) there is no clinical sign of peri-implantitis. The secondary objective of the present study was to demonstrate a non-inferiority of the HY-coated implants compared to the control implants considering the marginal bone resorption parameter. To do this, the null hypothesis is that control implants demonstrate an inferior crestal bone resorption in respect to HY coated implants.

##### Sample Size Calculation

The sample size was estimated based on the crestal bone level at 12 months post implant. 

Since the objective of this study was the non-inferiority of the investigational device (ID) compared to the Control ID in a crossover design from data published by Mumcu [82], inferior limits for the Control ID were estimated.

Practically, from pooling data reported in Table 1 of Mumcu and Coll., an inferior limit of 0.7 mm was estimated and considered clinically appropriate. This implies that if the lower bond of the 95% confidence interval for the estimated difference in crestal bone level between implants is above ‒0.7 mm, then the ID is considered non-inferior to the Control ID.

Sample size was calculated under the following assumptions:

- Lower limits in difference between implants: −0.70 mm (∆)
- Hypothesis testing:
- H0: µControl ID- µID ≤ ∆
- H1: µControl ID- µID > ∆ (ID implant is non-inferior with respect to the mean response)
- α level of probability: 0.025 for one-sided test
- 1-β (power) level: 90% for a conservative approach
- Standard deviation for mean difference: 0.66, which corresponded to 80% of the pooled mean calculated from Mumcu (0.83 mm). In this estimate, it was considered that the ID presented on the crestal bone level a greater variability in respect to the Control ID and that reported in the publication.
  By applying the formula reported in Julius [83] for non-inferiority trials in crossover design:
  $$n=\frac{2\sigma^{2}{\left(Z_{\left(1-\beta \right)}+Z_{\left(1-\alpha \right)}\right)}^{2}}{{\left(\left(\mu_{Control\;ID}-\mu_{ID}\right)-d\right)}^{2}}$$

18 evaluable subjects are needed to demonstrate the non-inferiority of the ID in respect to the Control ID. Considering that a couple of devices are implanted into each subject, we need to have at least 18 implant pairs in order to reach the trial primary objective.

## 5. Conclusions

The clinical trial “Blind Comparison of Covalently-Linked Hyaluronan versus Control-Dental Implants in a Randomized Crossover Clinical Investigation” evaluated clinical success up to 36 months with HY-coated dental implants compared to the control—uncoated microrough Ti grade 4 implants. Results showed a lack of differences between the two arms of the study. Both of them provided optimal healing. 

Substitution of the clinically accepted and consolidated titanium surface chemistry with the molecular structure of HY and ensuing interfacial interactions results in a satisfactory clinical outcome.

## Figures and Tables

**Table 1:** Roughness parameters obtained by stereo-SEM. Data are expressed in µm as average and standard deviation of three measurements.

Parameter	Control		HY-Coated		Description
	Mean	Std	Mean	Std	
Ra	0.97	0.19	0.93	0.24	Average roughness of profile
Rq	1.26	0.29	1.23	0.34	Root-Mean-Square roughness of profile
Rt	7.96	0.77	6.87	1.13	Maximum peak to valley height of roughness profile
Rz	5.67	1.23	5.77	1.15	Mean peak to valley height of roughness profile
Rmax	7.93	0.75	6.41	0.99	Maximum peak to valley height of roughness profile within a sampling length
Rp	4.33	0.63	3.93	0.73	Maximum peak height of roughness profile
Rv	3.63	0.14	2.93	0.45	Maximum valley height of roughness profile
Rc	3.69	0.64	3.62	1.03	Mean height of profile irregularities of roughness profile
Rsm	43.22	9.69	53.77	19.90	Mean spacing of profile irregularities of roughness profile

**Table 2:** Surface composition (% at.) of Control and HY-coated implants.

Sample	O	Ti	N	C
Control	51.4	17.1	1.0	30.5
HY-coated	28.7	-	7.9	63.4

**Table 3:** Demographic data and implant location. Data in absolute values and (%).

Demographic Data	Total	HY	C
Males	21 (70)		
Females	9 (30)		
Patients mean age	59.8±10.6		
**Implant location**			
Anterior maxilla	18 (17.0)	9 (16.4)	9 (17.6)
Posterior maxilla	41 (38.7)	24 (43.6)	17 (33.3)
Anterior mandibula	10 (9.4)	5 (9.0)	5 (9.8)
Posterior mandibula	37 (34.9)	17 (30.9)	20 (39.2)

**Table 4:** Bone resorption.

Time Points	HY		C		*p* (between-Group)	95% Confidence Interval	
	*n*	Mean ± sd	*n*	Mean ± sd		Min	Max
Mesial							
3	51	0.55 ± 0.46	47	0.51 ± 0.65	0.64	−0.24	0.55
6	24	0.72 ± 0.38	20	0.65 ± 0.39	0.58	−0.30	0.17
12	35	0.83 ± 0.61	35	0.66 ± 0.58	0.25	−0.45	0.12
18	16	0.65 ± 0.63	17	0.36 ± 0.43	0.14	−0.67	0.10
24	34	0.80 ± 0.87	32	0.50 ± 0.57	0.10	−0.66	0.06
36	21	0.55 ± 0.40	19	0.32 ± 0.36	0.06	−0.47	0.01
Distal							
3	51	0.71 ± 0.60	47	0.70 ± 0.62	0.97	−0.25	0.24
6	24	0.77 ± 0.60	20	0.93 ± 0.46	0.30	−0.15	0.49
12	35	0.83 ± 0.63	35	0.85 ± 0.60	0.90	−0.28	0.31
18	16	0.72 ± 0.53	17	0.47 ± 0.45	0.16	−0.59	0.11
24	34	0.76 ± 0.42	32	0.63 ± 0.62	0.33	−0.39	0.14
36	21	0.62 ± 0.53	19	0.55 ± 0.62	0.70	−0.44	0.30

Time points: months; *n*: number of implants; mean ± standard deviation (sd) expressed in mm.

**Table 5:** Criteria for inclusion in the clinical trial.

Inclusion Criteria
Over 18 years of age;
Bilateral loss of one or more molars and bicuspids and/or bilateral loss of anterior teeth and the need for more than one implant in the same rehabilitation;
Edentulousness of both upper or lower jaw with need of at least 2 bilateral implants to stabilize a denture or to support a fixed prosthesis;
In general good health condition and with physical ability to tolerate surgical and prosthetic procedure (ASA 1 and 2);
Good plaque control and oral hygiene;
The subject who agree to return to the center for follow-up.

**Table 6:** Criteria for exclusion from the clinical trial.

Exclusion Criteria
Active infection or severe inflammation or suspected lesions in the areas intended for implant installation;
Diabetes (regardless of control);
Need for concomitant bone grafting and/or having less than 1 mm bone available at the buccal, lingual, and apical aspects of the implant. Less than 3 mm distance between implant and other dentition;
Under treatment and/or within the past 12 months with radiotherapy to the head or chemotherapy;
Suspected hypersensitivity and/or contraindication to any ingredients of the Investigational Device (ID)/Control ID;
Subjects under any study medication treatment in the last 30 days;
Pregnancy, breast feeding, oocyte donation, or oocyte implantation planned during the study;
Subjects not able to follow study procedures, e.g., language problems, psychological disorders;
Clinically relevant abnormal laboratory values suggesting an unknown disease and requiring further clinical evaluation (as assessed by the investigators);
Female subjects of childbearing potential, not using and not willing to continue using a medically reliable method of contraception for the entire study duration;
Any other untoward medical condition that could interfere with the participation of the subject in the trial.

## Abbreviations

BMTiS	Biochemical modification of titanium surfaces
ECM	Extra Cellular Matrix
EDX	Energy Dispersive X-ray Analysis
GCP	Good Clinical Practice
HY	hyaluronic acid or hyaluronan
ID	Investigational Device
MAG	level of magnification
RCT	Randomized Clinical Trial
SEM	Scanning Electron Microscopy
XPS	X-ray Photoelectron Spectroscopy
